# Ammonia Exposure-Induced Immunological Damage in Chicken Lymphoid Organs via TLR-7/MYD88/NF-κB Signaling Pathway and NLRP3 Inflammasome Activation

**DOI:** 10.4014/jmb.2407.07025

**Published:** 2024-10-22

**Authors:** Mujeeb ur Rahman, Muhammad Wajid Ullah, Sehrish Manan, Hazrat Bilal, Thamir Alomairy, Mohamed F. Awad, Said Nawab, Zahoor, Daochen Zhu

**Affiliations:** 1 Biofuels Institute, School of Emergency Management, School of the Environment and Safety Engineering, Jiangsu University, Zhenjiang 212013, P.R. China; 2 Department of Pulp & Paper Engineering, College of Light Industry and Food Engineering, Nanjing Forestry University, Nanjing 210037, P.R. China; 3 Jiangxi Key Laboratory of Oncology, JXHC Key Laboratory of Tumour Metastasis, Jiangxi Cancer Hospital, The Second Affiliated Hospital of Nanchang Medical College, Jiangxi Cancer Institute, Nanchang, Jiangxi 330029, P.R. China; 4 Department of Biology, Faculty of Science, Umm Al-Qura University, Makkah 21955, Saudi Arabia; 5 Department of Biology, College of Science, Taif University, Taif 21944, Saudi Arabia; 6 Jiangsu Collaborative Innovation Center of Technology and Material of Water Treatment, Suzhou University of Science and Technology, Suzhou 215009, P.R. China

**Keywords:** Ammonia, bursa of fabricius, thymus, inflammasome, toll-like receptors, NF-κB

## Abstract

Ammonia (NH_3_) is a hazardous gas that pollutes the environment and causes irritation. Its harmful effects on chickens, including its impact on their immune system, have previously been observed. However, the mechanism by which NH_3_ exposure causes immune system disorders in chickens remains unclear. The bursa of Fabricius (BF) and thymus are the two main lymphoid organs responsible for the proliferation, differentiation, and selection of B- and T-lymphocytes, both critical for the innate immune response of the host. In this study, we investigated the mechanism of NH_3_-induced immune dysregulation in broiler chickens. Transmission electron microscopy (TEM) revealed swollen mitochondria and breakage of the large crista lining, membrane deformation, chromatin condensation, increased vacuolation, and blood vessel spasms in the NH_3_-exposed BF and thymus tissues. Immunofluorescent analysis showed clustering of CD4^+^ and CD8^+^ cells, indicating an active immune response to NH_3_ exposure. Furthermore, NH_3_ exposure enhanced the mRNA expressions of Toll-like receptor 7 (TLR-7), myeloid differentiation primary response 88 (MYD88), and nuclear factor-kappa B (NF-κB), along with their proteins, and led to activation of the TLR-7/MyD88/NF-κB signaling pathway and NLRP3 inflammasome in chicken thymus tissues. Both mRNA and protein levels of key inflammation-related genes and proteins were upregulated in the NH_3_-treated group, highlighting a robust inflammatory response due to NH_3_ exposure. The specific findings of significant structural damage to key lymphoid organs and activation of inflammatory pathways in broiler chickens upon NH_3_ exposure can provide guidance for future, targeted therapies to improve poultry health.

## Introduction

Ammonia (NH_3_) emitted by agriculture, particularly from poultry farms and barns, is a significant contributor to acid rain. Despite its prevalence, the impact of atmospheric NH_3_ on human health is under-researched and under-emphasized in the scientific literature [1, 2]. Existing studies indicate that NH_3_ can negatively affect lung function in individuals handling livestock [3], leading to decreased lung capacity, acute throat and eye irritation, and increased cough and phlegm production [4]. Moreover, recent findings suggest that agriculture NH_3_ emissions might contribute to childhood asthma and other respiratory conditions. A major concern with NH_3_ is its role in the formation of fine particulate matter (PM_2.5_), as it accounts for some 30% of this particulate fraction in America, and over 50% in Europe. PM_2.5_ particles can penetrate deep into the lung, causing chronic and fatal diseases, such as obstructive pulmonary disease, lung cancer, and inflammation fibrosis [5-7].

NH_3_ emissions can lead to immune dysfunction, potentially setting the stage for various diseases in humans and animals [8, 9]. However, the exact threshold at which NH_3_ exposure does not interfere with the immune system remains unknown. In poultry, NH_3_ exposure has been shown to impair the immune system. High NH_3_ levels can reduce the mass of lymphoid organs and cause inflammation in organs like the spleen [10]. Thymus and bursa of Fabricius (BF), key lymphoid organs in chicken, play crucial roles in cell-mediated and humoral immunity, respectively [11, 12]. Yet, the specific effects of NH_3_ on these organs are not fully understood.

The inflammasome, a cluster of Nod-like receptors (NLR), functions as an intracellular protein sensor and is crucial for immune response. Dysfunction in inflammasome components, particularly the NLRP3 receptor, can lead to severe immune disorders [13-15]. NLRP3 activation can be triggered by reactive oxygen species (ROS), pathogen-associated molecular patterns (PAMPs), and damage-associated molecular patterns (DMAPs) [16]. Although NLRP3 activation has been observed in chicken lungs, there is limited research on its activation in lymphoid organs due to NH_3_ inhalation [17]. Research indicates that the nuclear factor-kappa B (NF-κB) pathway plays a significant role in promoting inflammation [18, 19]. Other studies have shown that rats exposed to NH_3_ develop liver inflammation via the NF-κB pathway [20]. NF-κB is known to enhance the production of cytokines, such as TNF-α, IL-1β, IL-10, and Cox-2, which are crucial for inflammatory responses [21]. Elevated levels of inducible nitric oxide synthase (iNOS) and nitric oxide (NO) further contribute to inflammation in various tissues [22]. However, the activation of the inflammasome in chicken lymphoid organs due to NH_3_ exposure remains under-investigated.

Here, we sought to elucidate the molecular pathways underlying NH_3_-induced immunological damage in broiler chicken lymphoid organs, focusing on the activation of the NLRP3 inflammasome and the TLR-7/MYD88/NF-κB signaling pathway. We investigated the mechanism of NH_3_-induced immune dysregulation in chickens by examining structural damage and inflammatory pathway activation in the BF and thymus. Transmission electron microscopy (TEM) was used to observe structural changes in BF and thymus tissues, whereas immunofluorescent analysis was carried out to detect the clustering of CD4+ and CD8+ cells. Additionally, the expression of mRNA and protein levels of TLR-7, MYD88, NF-κB, and NLRP3 inflammasome were determined to evaluate the activation of inflammatory pathways. This study can provide both a better understanding of the mechanism of NH_3_ toxicity and a basis for future therapeutic studies aimed at controlling the toxic effects of NH_3_.

## Materials and Methods

### Preparation of Broiler Chickens for Experimentation

We acquired 60, 1-day-old broiler chicks (Ross 308) from Weiwei Co. Ltd. (China). The broilers were kept in a controlled environment chamber. The temperature, humidity, and light levels were set in accordance with the Ross broiler handbook, and the chicks were randomly divided in two groups of 30 per set (control and NH_3_-treated (45.0 mg/m^3^). The chicks in the non-treated group were not exposed to NH_3_. A compressed anhydrous NH_3_ gas cylinder (Dawn Gas Co. Ltd., China) was used to adjust the NH_3_ content, which was then monitored with a Photoacoustic Field Gas-Monitor Innova-1412 (Lumasense Technologies, Inc., USA). Environmental parameters such as temperature, humidity, and light were monitored, as reported previously [8]. The study was approved by the Institutional Animal Care and Use Committee of Jiangsu University (Zhenjiang, China) (Approval ID: JU-20240005599).

### Sample Collection

On the 42nd day, ten broilers from each group were randomly chosen and sacrificed using cervical dislocation. Bursa of Fabricius (BF) and thymus samples were taken and washed with ice-cold phosphate-buffered saline (PBS, pH 7.2). The samples were then immersed in cold, deionized water for further processing before being stored at -80°C.

### Immunofluorescence Analysis

For the immunofluorescence (IF) experiment, Luminol was applied to 5-micron-thick tissue sections for 15 min. In 75% and 85% ethanol, the sections were dehydrated every 5 min and then washed with deionized water. Afterwards, to isolate antigens, the samples were soaked in a boiling EDTA solution with a pH of 8.0 for 7-8 min. The samples were cooled for 5 min in a PBS solution (pH 8.0) in a rocker device to prevent evaporation of the buffer solution. A liquid block marker pen was used to prevent liquid from leaking. The samples were then immersed in a spontaneous fluorescence quenching solution for 5 min before being washed with fresh water and incubated at room temperature with primary antibody diluted in goat serum PBS. Anti-CD8α antibodies (diluted 1:500, Biomos Technology Co. Ltd., China) were added to the blocking solution and incubated overnight at 4°C. The cells were then stained with FITC-conjugated anti-rabbit IgG (diluted 1:400; Wuhan Servicebio Technology Co. Ltd., China) and placed in DAPI-containing media. Finally, the slides were inspected with an inverted microscope (Nikon TE2000, Nikon Co. Ltd., Japan).

### Transmission Electron Microscopic Observation

The following protocol was used for transmission electron microscopy (TEM) observations. First, the samples were fixed with 2.5% glutaraldehyde. Afterwards, each aliquot of the fixed sample was rinsed three times in phosphate-buffered saline (PBS), each for 5 min. After this step, the samples were immersed in a 1% osmium tetroxide buffer solution for 60 min to achieve an osmium bronze coating. The specimens were dehydrated and embedded in epoxy resin. Next, a thinly sliced sample was stained with uranyl acetate and briefly rinsed in water for 5 s on either side. The sample was then used as a substrate for transmission electron microscopy observation (TEM, Hitachi 7650S, Japan).

### RNA Extraction and Real-Time Qunatitative PCR

Following RNA extraction in accordance with Shah *et al*. 2020, real-time quantitative PCR (qPCR) analysis was conducted. RNA from each animal was separately prepared. After being homogenized in Tri-Reagent, the RNA of the BF and thymus tissues was extracted according to the manufacturer's instructions. The extracted RNA purity and concentration were tested at 260/280 nm absorbance with a Gene Quan Vevo 1300/100 spectrophotometer (China).

### Complementary DNA Synthesis

The procedure for the synthesis of cDNA was as follows: for the reverse transcription of 3.0 μl of RNA, 3.0 μl of Golden M-MLVIII Reverse Transcriptase, 6.0 μl of 10 RT Buffer, 3.0 μl of dNTP combination of 10 mM of each nucleotide, and 3.0 μl of 20 × oligo dT25 primer were added, as well as 1.5 μl of RNase inhibitor and 43.5 μl of RNase-free water. Based on the procedure, the reaction mixture was first incubated at 30°C for 15 min, then at 55°C for 50 min, and finally at 80°C for 10 min, to create cDNA. The produced cDNA was reserved for use in qRT-PCR experiments, and kept at -20°C at a 500-fold greater concentration. Also shown below in Table 1 are the internal control genes with the specific gene primer sets.

### Preparation of Master Mix

The samples were prepared in 5 μl of 2× SYBR Green PCR Master Mix, 0.3 μl forward and reverse primers, 3.4 μl sterile distilled water, and 1 μl cDNA. The qRT-PCR was followed by thermal cycling settings as follows: an initial denaturation at 95°C for 10 min; then 40 cycles of denaturation for 15 s, with annealing/extension for 60 s, which was prolonged for less than 20 s. Three attempts were carried out for each sample, and the results in terms of relative mRNA levels were presented according to a previously reported method [23].

### Western Blot Analysis

The protocol described by Shah et al. was used for western blotting analysis, and 8-12% gradient gels were used to separate the proteins. Protein transfer was carried out using Pall Corporation PVDF membranes, which were blocked with 5% skim milk at 37°C for 1 h before being washed in buffer. The primary antibodies and a β-actin loading control antibody for membranes (Bioss Antibodies Inc., China) were then incubated overnight at 4°C. Before incubation, membranes with secondary antibodies (goat anti-rabbit or anti-mouse horseradish peroxidase-conjugated IgG) (Bioss Antibodies Inc.) at a dilution of 1:3000 were washed thrice using Tris-Buffered Saline with Tween (TBST). Protein bands were detected using Biosharp Life Sciences' chemiluminescence reagent (ECL) [8]. The proteins were loaded on a 12% SDS-PAGE system for separation, and then shifted to nitrocellulose membranes. After being blocked with 5% non-fat milk in TBST for 2 h and incubated with diluted primary chicken antibodies, including TLR-7 (1:500), MyD88 (1:1,000), TRAF6 (1:1,000), NF-κB (p65) (1:500), NF-κB (p-p65) (1:1,000), IκBα (1:500), and p-IκBα (1:1,000) as well as NLRP3, Pro-caspase-1, Caspase-1, and IL-18 at 1 h for 37°C, the membranes were washed four times with PBST for 5 min each time. Then, they were incubated in horseradish peroxidase-conjugated secondary antibodies against rabbit IgG (1:2000; Santa Cruz, Santa Cruz Biotechnology, Inc., USA) at 37°C for 1 h. Enhanced chemiluminescence (ELC, Biosharp Life Sciences, China) and ImageJ software (National Institute of Health, USA) were used for the visuals of the bound immune complexes.

### Statistical Analysis

One-way ANOVA was used to determine the statistical significance (*p* < 0.05) among experimental groups at the same time point and among different time points in the same group, followed by the least significant difference test through the statistical package for social science (SPSS Windows version 21.0, IBM, USA). GraphPad Prism software (Windows version 6.01; GraphPad Software, Inc., USA) was used to make all the graphs, and data were expressed as mean 6 SD.

## Results

### Transmission Electron Microscopic Observation of Lymphoid Organs

**Fig. 1:**
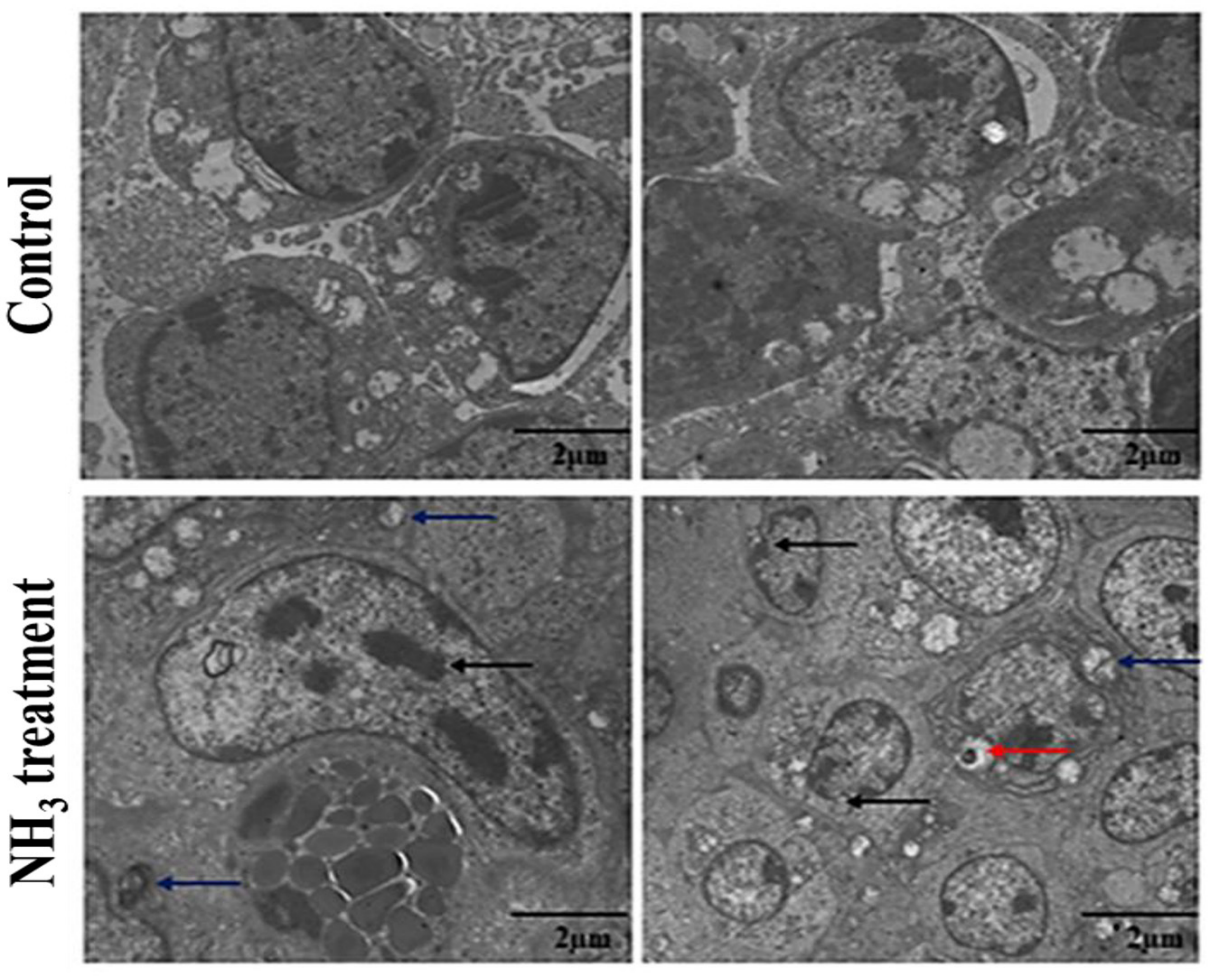
Transmission electron microscopic observation of thymus after 42 days of NH_3_ exposure. (**A**) Nontreated (control) group: This image shows healthy thymus cells with normal structures and serves as the baseline for comparison. (**B**) NH_3_-treated group: The red arrows indicate deformed cells caused by exposure to NH_3_, showing signs of damage. Blue arrows highlight swollen mitochondria, which suggests the disruption of the cell energy production process. Experiments were performed in triplicate (*n* = 3).

TEM observation showed that thymus tissues in the control group exhibited a typical ultrastructure with no significant signs or pathological changes (Fig. 1). The cells possessed healthy and undamaged mitochondria in the control group, and were characterized by well-preserved cristae and intact structures, indicating normal cellular respiration and energy production processes [23]. The cells in the control group also showed intact cell membranes with no sign of deformation or damage, suggesting that the cells were maintaining their structural integrity and not undergoing stress or damage. In contrast, the cells in the NH_3_-treated group exhibited swollen mitochondria, indicating mitochondrial dysfunction due to NH_3_-induced stress. This can affect the ability of mitochondria to produce ATP efficiently, leading to cellular energy deficits. Likewise, the cells in the NH_3_-treated group exhibited deformation in their cell membranes, indicative of compromised cellular integrity and potential damage to the external protective barrier. This membrane deformation may allow the influx of harmful substances and loss of essential cellular components, exacerbating cellular stress. The cells in the NH_3_-treated group also showed increased vacuolation and changes in the intracellular substance (indicated by black arrows), suggesting increased autophagy or lysosomal activity as the cells attempt to manage and recycle damaged components. Blood vessel spasms are also evident, which are likely caused by nuclear breakdown and cellular stress responses. These spasms could disrupt normal blood flow and nutrient delivery, further impacting cellular health. Sequential nuclear breakdown, which may further lead to cellular and structural damage, was also observed [24, 25]. TEM observation of the cells in the NH_3_-treated group suggests that the cellular damage is not limited to the cytoplasm and mitochondria but extends to the nucleus, potentially affecting DNA integrity in cellular replication. Overall, the ultrastructural analysis of the control and NH_3_-treated groups revealed significant differences in cellular health and integrity. While the control group maintained normal cellular architecture with healthy mitochondria and intact cell membranes, the NH_3_-treated group exhibited substantial pathological changes. These changes include mitochondrial swelling, cell membrane deformation, increased vacuolation, and blood vessel spasms, all indicative of severe cellular damage and stress due to NH_3_ exposure. These findings show the toxic impact of NH_3_ on cellular structures and highlight the importance of maintaining a healthy cellular environment to prevent such damage.

**Fig. 2:**
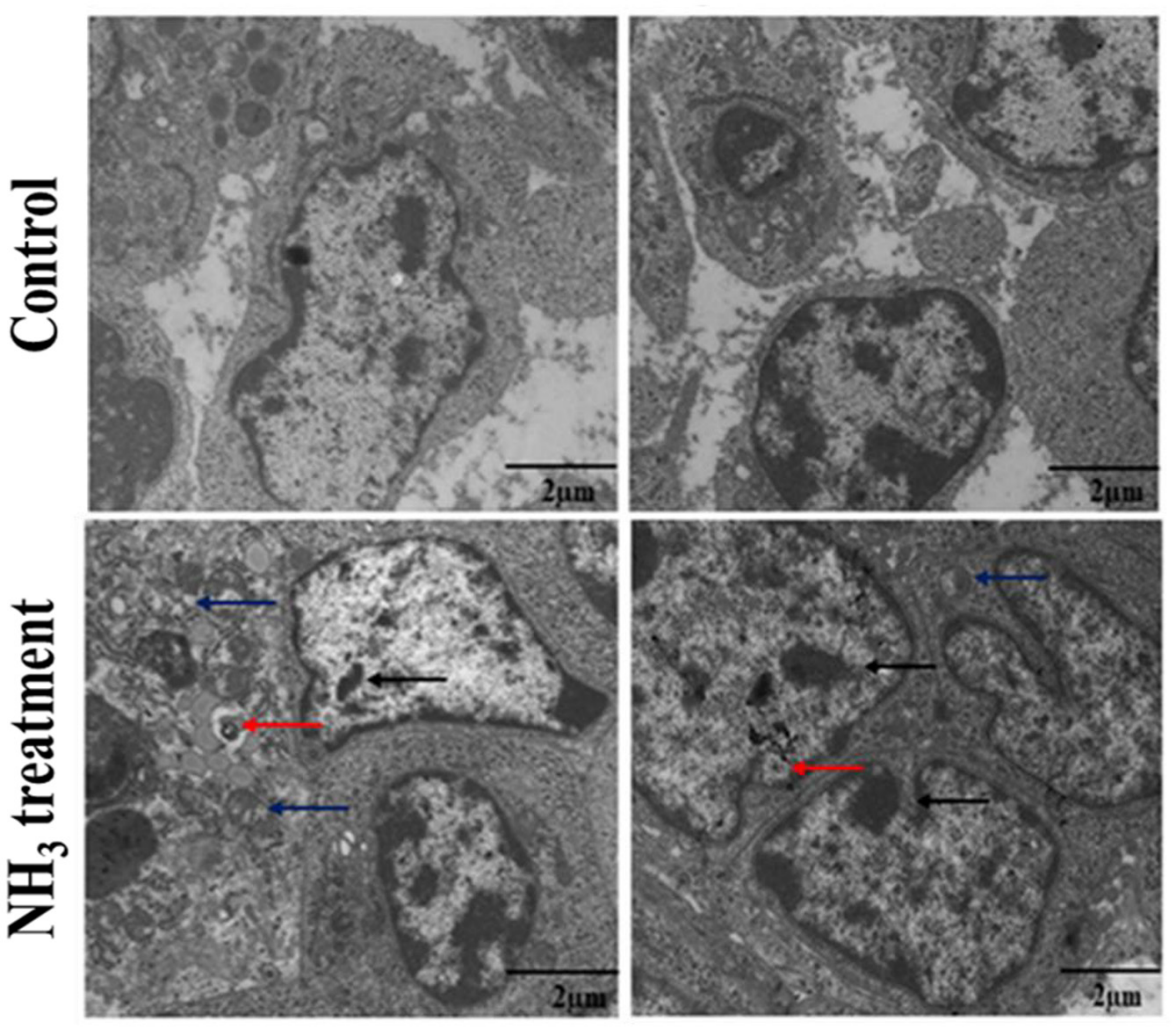
Transmission electron microscopic observation after 42 days demonstrating the influence of NH_3_ on the chicken bursa of Fabricius (BF). (**A**) Non-treated (control) group: This image shows a healthy chicken BF with normal structures and serves as the baseline for comparison. (**B**) NH_3_-treated group: The blue arrows show mitochondrial swelling, while the red arrows show broadened intercellular areas, nuclear lysis, and cell membrane distortion. Experiments were performed in triplicate (*n* = 3).

TEM observation shows intact and clear mitochondria with well-defined cristae and preserved BF structure in the control group (Fig. 2), indicating healthy cells that are maintaining mitochondrial function and energy production. The cells also exhibited intact cell membranes with no signs of distortion or damage, suggesting well-maintained cellular integrity and no evidence of stress or pathological changes. Overall, the cells in the control group show normal ultrastructural appearance with typical organelle distribution and no apparent signs of cellular distress. In contrast, the cells in the NH_3_-treated group show significant mitochondrial swelling, indicating that the dysfunction is likely due to the toxic effects of NH_3_ exposure, which may lead to impaired mitochondrial respiration and ATP synthesis. The cells also showed a noticeable broadening of intracellular areas, suggesting edema or accumulation of extracellular fluid. This broadening is indicative of disrupted cell-cell communication and potential loss of cohesion. The cells showed nuclear lysis, which may lead to loss of genetic material and impaired cellular function, ultimately resulting in cell death. The cells also showed significant distortion of the cell membrane (red arrows), suggesting compromised cellular integrity, which may allow the entrance of harmful substances and leakage of cellular components. All these events indicate severe cellular stress and pathology leading to impaired function or cell death. Overall, the ultrastructural comparison through TEM between the control and NH_3_-treated groups highlights significant differences in cellular health and integrity. While the control group maintains normal cellular architecture with intact mitochondria and membranes, the NH_3_-treated group shows severe pathological changes, all indicative of the toxic effects of NH_3_ exposure. These findings suggest the detrimental impact of NH_3_ on the cellular structures of the chicken BF, emphasizing the importance of maintaining a healthy environment to prevent such damage.

### Level of CD4+ and CD8+ Lymphocytes in Thymus and BF

**Fig. 3:**
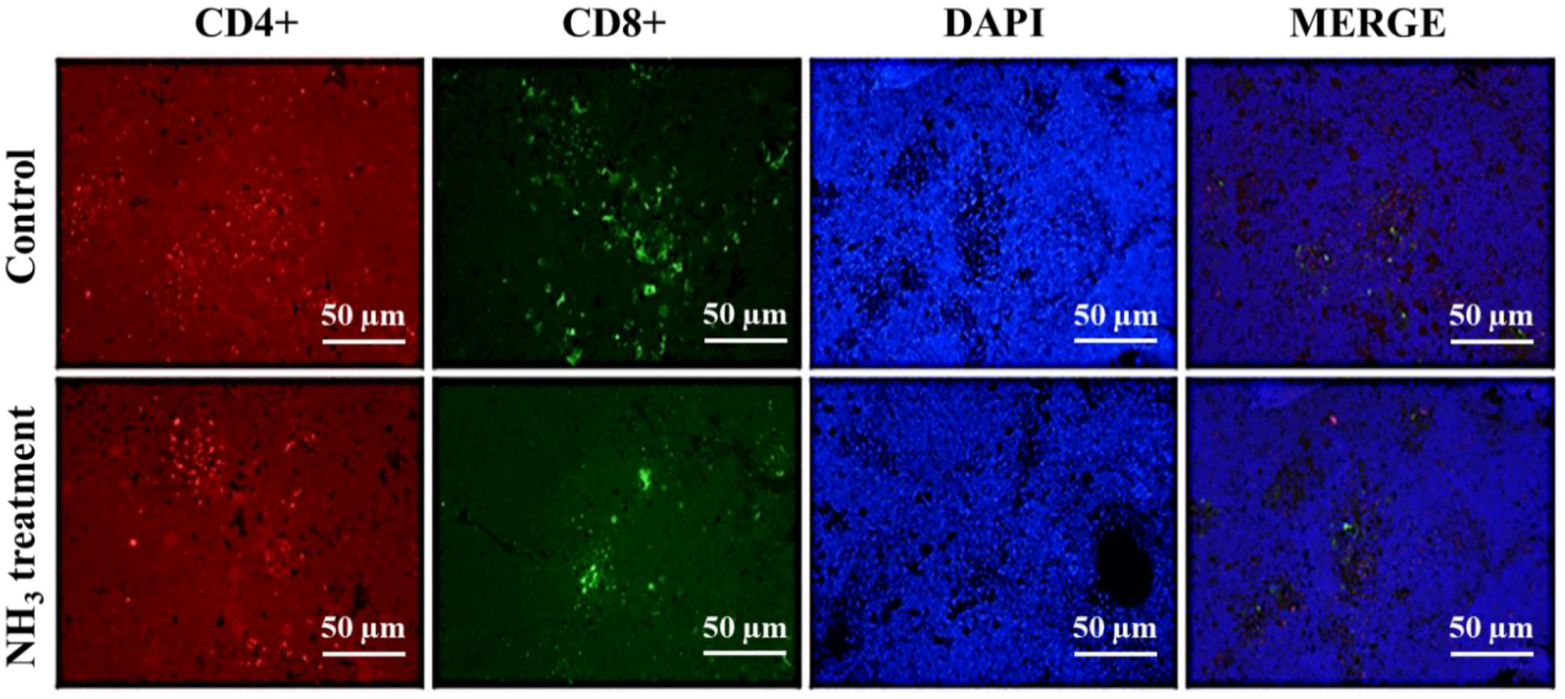
The immune cell profiles from thymus tissues of control and NH_3_-treated groups using an experimental NH_3_ emission model. The immune cells are visualized using immunofluorescence staining. Fluorescent marks for CD4^+^ (red), CD8^+^ (green), and DAPI (blue) are used to stain cellular nuclei. Images were captured at 400x magnification for both non-treated and NH_3_-treated groups (*n* = 3). The immunofluorescent micrographs in each group are labeled CD4+, CD8+, DAPI, and Merge.

The immunofluorescent image in the control group shows the presence of well-dispersed CD4+ cells (red-stained), indicating a normal distribution of these helper T cells in the thymus (Fig. 3). The CD8^+^ cells (green-stained) show normal distribution with a moderate presence throughout the tissue. DAPI staining shows evenly distributed nuclei (blue-stained), suggesting normal cell density and nuclear integrity. The merged images of CD4^+^, CD8^+^, and DAPI-stained cells show the spatial relationship between CD4^+^ and CD8^+^ cells and their nuclei. In contrast, immunofluorescent images in the NH_3_-treated group show a slight increase or clustering of CD4^+^ cells (red-stained) in some places, suggesting a potential immune response to NH_3_ exposure. The CD8^+^ cells (green-stained) also show a noticeable increase in their numbering and clustering, indicating an increased cytotoxic T-cell response due to NH_3_-induced stress. While the distribution of nuclei remains relatively normal (DAPI staining), there are some areas of increased cell density that suggest potential cellular proliferation or immune cell infiltration. The merged image shows the increased presence and clustering of immune cells (CD4^+^ and CD8^+^) around the nuclei, suggesting an active immune response to NH_3_ exposure [24]. In summary, the immunofluorescence analysis of the control and NH_3_-treated groups reveals significant differences in immune cell profiles. The control group displays a normal distribution of CD4+ and CD8+ cells, with evenly distributed nuclei. In contrast, the NH_3_-treated group shows an increased presence and clustering of CD8^+^ cells and a slight increase in CD4^+^ cells, indicating an active immune response to NH_3_ exposure. The DAPI staining suggests that nuclear integrity and cell density are relatively maintained, although there may be areas of increased cellular activity. These findings show that NH_3_ exposure induces a significant immunological response, potentially impacting thymus development and function.

**Fig. 4:**
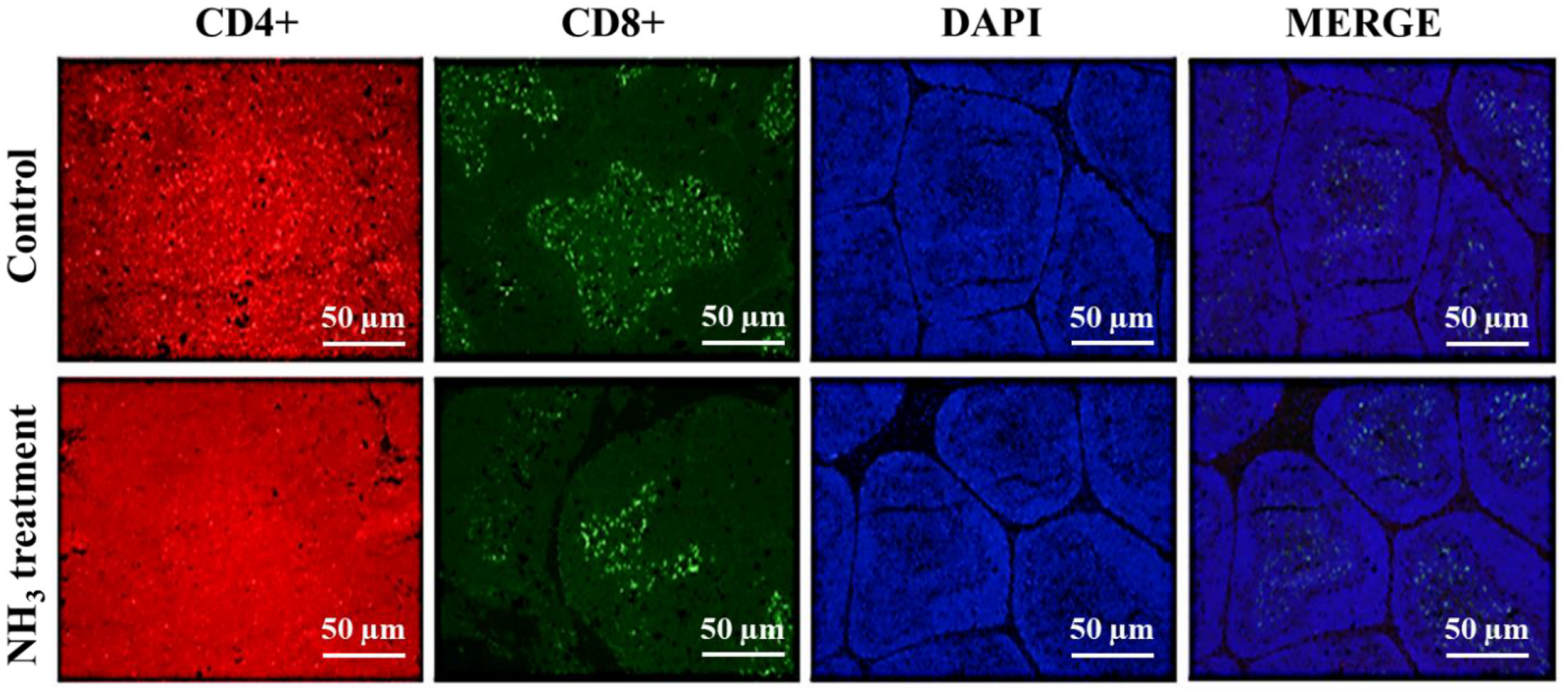
Estimation of CD4+ and CD8+ cells in the BF of broilers using immunofluorescent microscopy. Images were captured at 400x magnification for both control and NH_3_-treated groups (*n* = 3). The fluorescent microscopy images are labeled as CD4+ (red), CD8+ (green), DAPI (blue), and merge, illustrating the distribution and density of these immune cells and nuclei in the two groups.

The immunofluorescent image in the control group shows uniformly distributed CD4^+^ cells (red-stained) throughout the FB tissue, indicating a normal presence of T helper cells in the broilers’ tissues (Fig. 4). The CD8^+^ cells (green-stained) show distinct clustering in certain regions, suggesting the typical location of cytotoxic T cells. DAPI staining (blue-stained) shows a uniform distribution of nuclei, suggesting normal cell density and nuclear integrity. The merged image shows the spatial relationship between CD4^+^ and CD8^+^ cells and their nuclei, illustrating the normal distribution and interaction of these immune cells in the control group. In contrast to the control group, a noticeable difference was observed in the distribution of CD4^+^ cells (red-stained) in the NH_3_-treated group, potentially indicating a response to NH_3_ exposure. CD8+ cells (green-stained) also appeared reduced in number, with fewer clusters compared to the control group, suggesting potential immunosuppression or cellular damage due to NH_3_ exposure. While DAPI staining (blue) shows a relatively uniform distribution of cells, there may be slight changes in cell density, indicating potential cellular stress or damage. The merged image also showed altered distribution and density of immune cells in response to NH_3_ exposure, indicating potential immunological impact and cellular stress. Overall, the immunofluorescence analysis of the control and NH_3_-treated groups reveals significant differences in the distribution and density of immune cells. The control group shows a normal presence and distribution of CD4^+^ and CD8^+^ cells with uniformly distributed nuclei. In contrast, the NH_3_-treated group exhibits a change in the distribution of CD4+ cells and a reduced density of CD8^+^ cells, suggesting an altered immune response and potential cellular stress due to NH_3_ exposure. The DAPI staining indicates that nuclear integrity is maintained, though there may be slight changes in cell density. These findings highlight the immunological impact of NH_3_ exposure on broilers, emphasizing the importance of maintaining a healthy environment to prevent such damage.

### Ammonia Activates NLPR3 Through TLR-7/MYD88/NF-κB

**Fig. 5:**
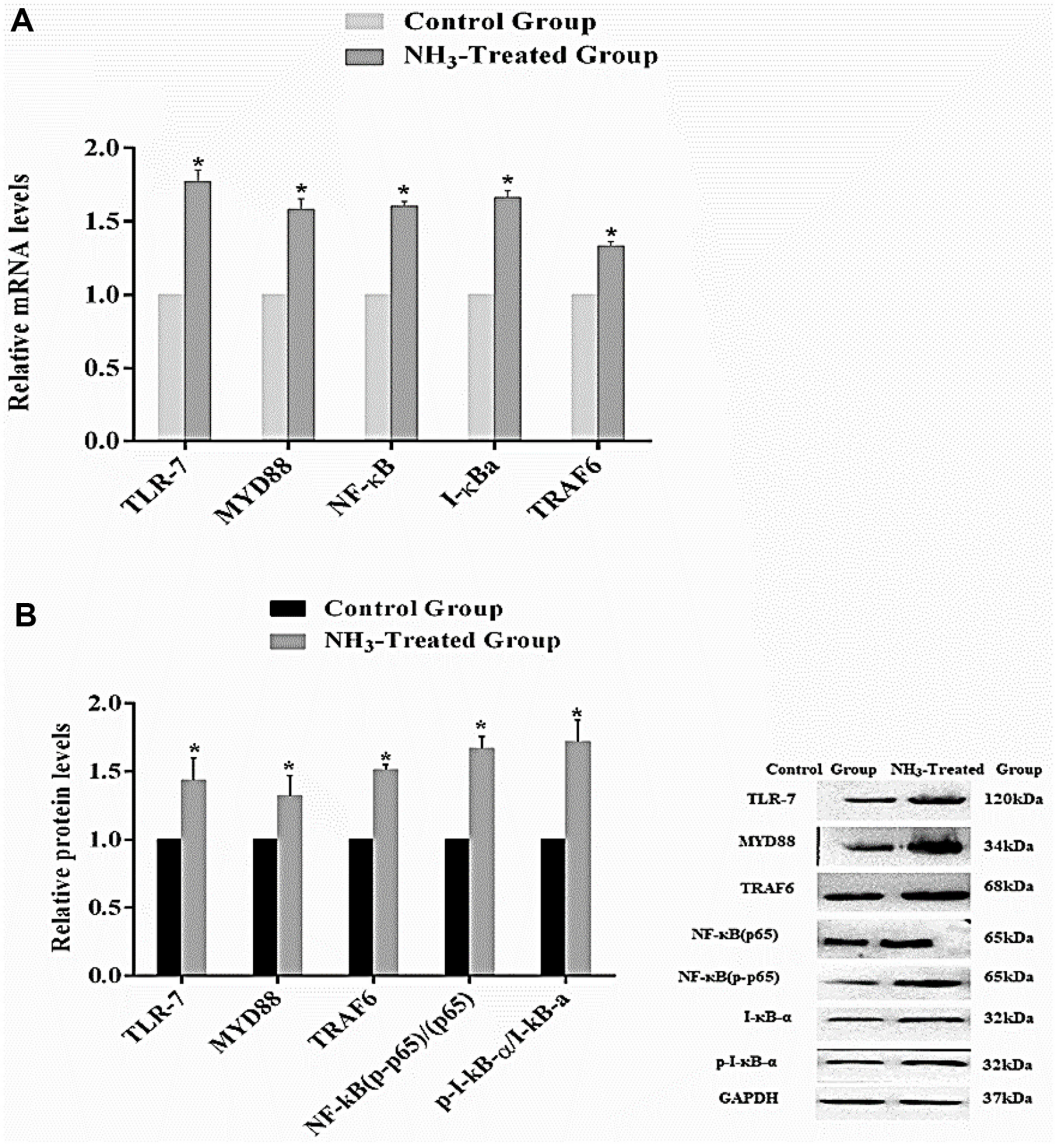
Activation of TLR-7/MyD88/NF-κB signaling pathway in chicken thymus tissues in control and NH_3_- treated groups. (**A**) mRNA levels of inflammation-related genes. (**B**) Protein expression level. Results are represented as bar graphs with the SD (*n* = 3). *p < 0.05 indicates a significant difference from the control group.

The relative mRNA levels of genes TLR-7, MyD88, NF-κB, IκBα, and TRAF6 in control and NH_3_-treated groups are shown in Fig. 5A. The results show that for each analyzed gene, the NH_3_-treated group shows a significant increase in mRNA levels compared to the control group (*p* < 0.05). The relative protein levels of TLR-7, MyD88, TRAF6, NF-κB (p65), NF-κB (p-p65), IκBα, and p-IκBα in both control and NH_3_-treated groups are shown in Fig. 5B. The results show that similar to mRNA levels, expression levels of all proteins under investigation are significantly higher in the NH_3_-treated group compared to the control group (*p* < 0.05). The lower levels of IκBα in the NH_3_-treated group are consistent with the activation of the NF-κB pathway, as it is typically degraded upon pathway activation. The western blot analysis further confirmed that NH_3_ exposure activated the TLR-7/MyD88/NF-κB signaling pathway at the protein level. Overall, the results show that NH_3_ exposure significantly activated the TLR-7/MyD88/NF-κB signaling pathway in chicken thymus tissues. Both mRNA and protein levels of key inflammation-related genes and proteins are upregulated in the NH_3_-treated group compared to the control group. The consistent increase in both mRNA and protein expression levels, alongside the degradation of IκBα, highlights the activation of this inflammatory signaling pathway due to NH_3_ exposure [17]. These findings suggest that NH_3_ exposure induces a robust inflammatory response in chicken thymus tissues, mediated through the TLR-7/MyD88/NF-κB signaling pathway.

**Fig. 6:**
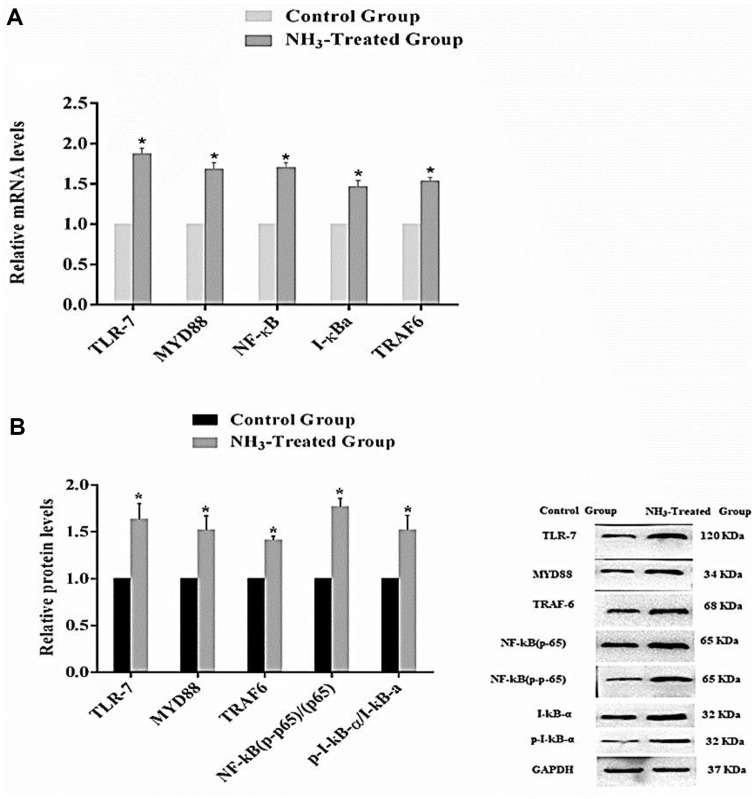
Activation of the TLR-7/MyD88/NF-κB signaling pathway in chicken BF tissues after NH_3_ exposure. (**A**) mRNA levels of inflammation-related genes. (**B**) Protein expression level. Results are represented as bar graphs with (SD) provided (*n* = 3). *p < 0.05 represents a significant difference compared to the control group.

The relative mRNA levels of the genes TLR-7, MyD88, NF-κB, IκBα, and TRAF6 are shown in Fig. 6A. The results show that for each gene analyzed, the NH_3_-treated group shows a significant increase in mRNA levels compared to the control group (*p* < 0.05). These results indicate that the exposure of BF tissues to NH_3_ significantly upregulated the expression of the inflammation-related genes at the mRNA level. The relative expressions of TLR-7, MyD88, TRAF6, NF-κB (p65), NF-κB (p-p65), IκBα, and p-IκBα proteins are shown in Fig. 6B. The results show that similar to mRNA levels, expression levels of all proteins are significantly higher in the NH_3_-treated group compared to the control group (*p* < 0.05). The lower levels of IκBα in the NH_3_-treated group are consistent with the activation of the NF-κB pathway, as it is typically degraded upon pathway activation. The western blot analysis provided visual confirmation of the differences in protein expression between the control and NH_3_-treated groups. This further confirms that NH_3_ exposure activated the TLR-7/MyD88/NF-κB signaling pathway at the protein level and is consistent with the protein expression in chicken thymus tissues. Overall, the results show that NH_3_ exposure significantly activated the TLR-7/MyD88/NF-κB signaling pathway in chicken BF tissues. Both mRNA and protein levels of key inflammation-related genes and proteins are upregulated in the NH_3_-treated group compared to the control group. The consistent increase in both mRNA and protein expression levels, alongside the degradation of IκBα, highlights the activation of this inflammatory signaling pathway due to NH_3_ exposure. These findings suggest that NH_3_ exposure induces a robust inflammatory response in chicken BF tissues, mediated through the TLR-7/MyD88/NF-κB signaling pathway.

**Fig. 7:**
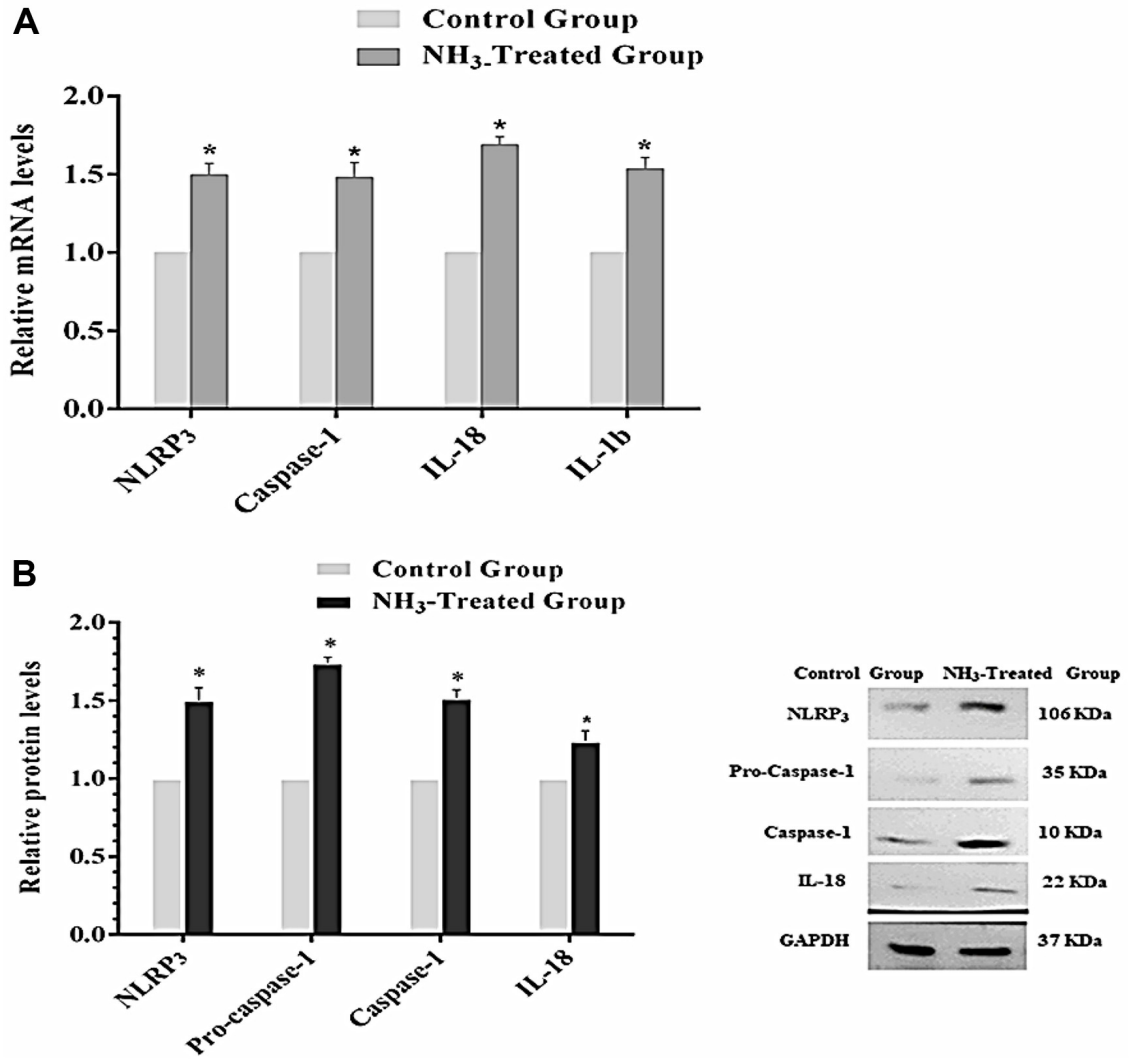
Activation of the NLRP3 inflammasome in chicken thymus after exposure to NH_3_. (**A**) mRNA levels of related genes. (**B**) Protein expression levels. Results are represented as bar graphs with SD provided (*n* = 3). *p < 0.05 represents a statistically significant difference from the non-treated (control) group.

The relative mRNA levels of the NLRP3, Caspase-1, IL-18, and IL-1β are shown in Fig. 7A. The results show that the NH_3_-treated group exhibited a significant increase in mRNA levels compared to the control group (*p* < 0.05). These results show that NH_3_ exposure significantly upregulated the expression of these inflammasome-related genes at the mRNA level [26]. Similar to mRNA levels, expression levels of NLRP3, Pro-caspase-1, Caspase-1, and IL-18 proteins are significantly higher in the NH_3_-treated group compared to the control group (*p* < 0.05)(Fig. 7B). The protein expression results are also in accordance with the visual expression determined through western blot analysis, in which GAPDH was used as a loading control to ensure equal protein loading across samples. Western blot also confirmed that NH_3_ exposure activated the NLRP3 inflammasome at the protein level. Overall, the results demonstrate that NH_3_ exposure significantly activated the NLRP3 inflammasome in chicken thymus tissues. Both mRNA and protein levels of key inflammasome-related genes and proteins are upregulated in the NH_3_-treated group compared to the control group. The consistent increase in both mRNA and protein expression levels highlights the activation of this inflammatory pathway due to NH_3_ exposure. These findings suggest that NH_3_ exposure induces a robust inflammatory response in chicken thymus tissues, mediated through the activation of the NLRP3 inflammasome. This underscores the potential immunological impact of NH_3_ and the importance of maintaining a safe environment for poultry to prevent such adverse effects.

**Fig. 8:**
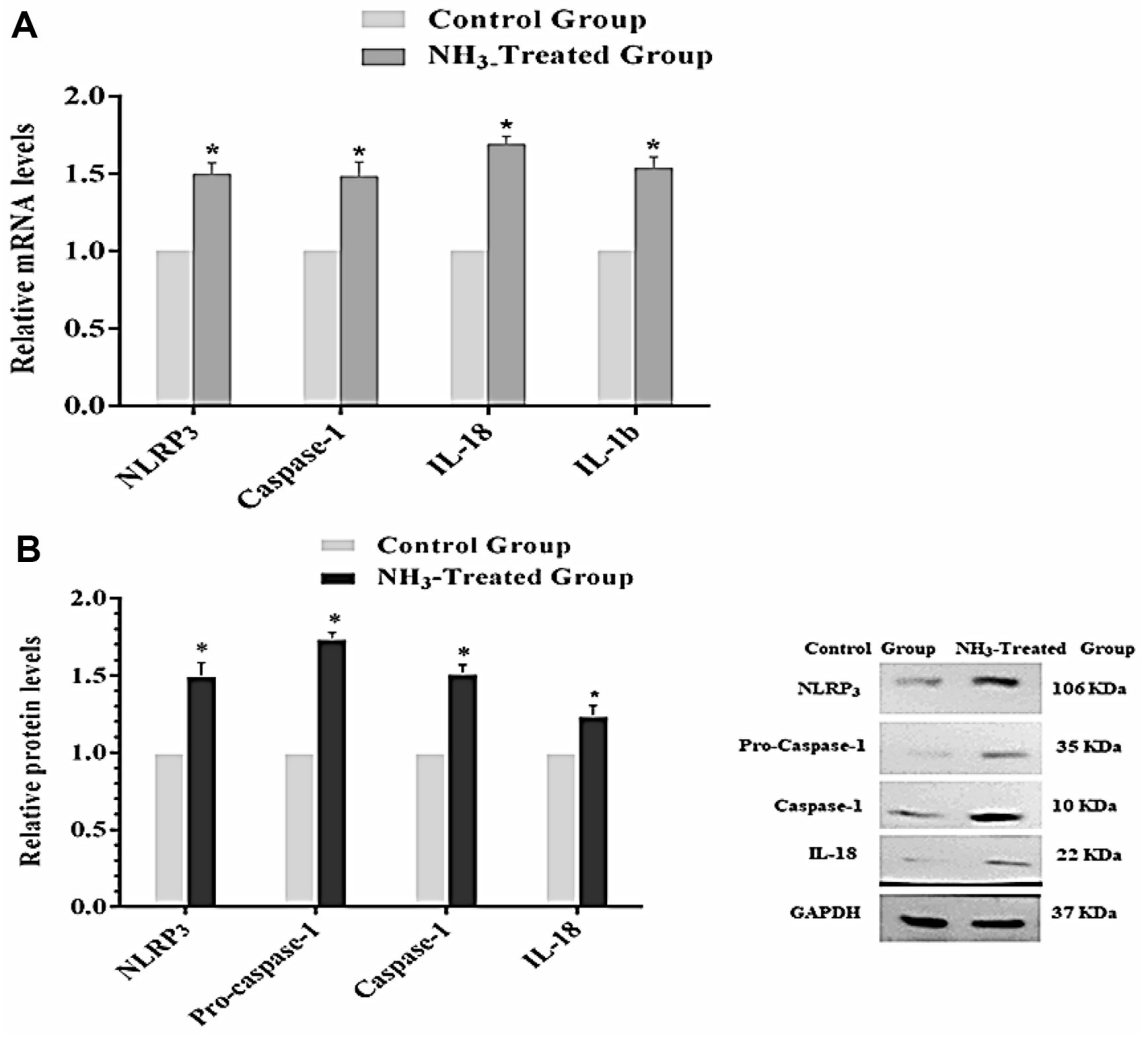
Activation of the NLRP3 inflammasome in chicken BF upon exposure to NH_3_. (**A**) mRNA levels of related genes. (**B**) Protein expression levels. Results are represented by bar graphs with the SD provided (*n* = 3). *p < 0.05 indicates a statistically significant difference between the NH_3_-treated and control groups.

The results of the relative mRNA levels of the NLRP3, Caspase-1, IL-1β, and IL-18 genes are shown in Fig. 8A. The results show that for each gene analyzed, the NH_3_-treated group showed a significant increase in mRNA levels compared to the control group (*p* < 0.05). This indicates that NH_3_ exposure significantly upregulated the expression of these inflammasome-related genes at the mRNA level. Similar to mRNA levels, expression levels of NLRP3, Pro-caspase-1, Caspase-1, and IL-18 proteins are significantly higher in the NH_3_-treated group compared to the control group (*p* < 0.05) (Fig. 8B). Western blot analysis shows a visual presentation of protein expression and further confirmed its upregulation after exposure to NH_3_, which caused activation of the NLRP3 inflammasome at the protein level. In summary, the results show that NH_3_ exposure significantly activated the NLRP3 inflammasome in chicken thymus tissues. Both mRNA and protein levels of key inflammasome-related genes and proteins are upregulated in the NH_3_-treated group compared to the control group. The consistent increase in both mRNA and protein expression levels highlights the activation of this inflammatory pathway due to NH_3_ exposure [27]. These findings suggest that NH_3_ exposure induces a robust inflammatory response in chicken thymus tissues, mediated through the activation of the NLRP3 inflammasome. This underscores the potential immunological impact of NH_3_ and the importance of maintaining a safe environment for poultry to prevent such adverse effects.

## Discussion

Ammonia exposure causes significant immune system disturbances in broiler chickens by reducing CD4^+^ and CD8^+^ lymphocytes and inducing ultrastructural damage in the BF and thymus. The current study revealed that NH_3_ exposure activated the TLR-7/MYD88/NF-κB signaling pathway, leading to the activation of NLRP3 inflammasomes. These findings suggest that NH_3_ disrupts immune regulation by provoking inflammatory responses. Our study further elucidated the mechanisms by which NH_3_ exposure leads to immune system disorders in broiler chickens, highlighting the activation of specific inflammatory pathways. These findings also provide a foundation for developing targeted therapies to mitigate the adverse effects of NH_3_ on poultry health, and thereby improve animal welfare and productivity in the poultry industry.

Lymphoid organs, the primary location for T and B-cell activation, are where proliferation and differentiation occur. CD4^+^ and CD8^+^ positive cells make up the vast majority of the lymphocyte population. These double-positive cells undergo a complex process of selection and recruitment. A study suggests that this form of selection occurs through the invasion of other pathogen infections and toxic gases [24]. Lymphoid organs are known to fight infections and control immune responses. Therefore, the effect of NH_3_ on these organs in broiler chickens was evaluated in this study. We found that a reduced quantity of CD4+ and CD8+ cells was observed by immunofluorescence assay in the NH_3_-treated group. TEM and immunofluorescence investigation provided significant insights into the cellular and immunological impacts of NH_3_ exposure on lymphoid organs, particularly in the BF and thymus. TEM observations revealed substantial differences in the ultrastructure of cells between the control and NH_3_-treated groups. The NH_3_-teated group exhibited significant pathological changes, including swollen mitochondria and mitochondrial dysfunction. This is consistent with previous studies showing that NH_3_ can induce mitochondrial swelling and disrupt mitochondrial function, leading to impaired ATP production [24]. The deformation of cell membranes observed in the NH_3_-teated group further supports the hypothesis of compromised cellular integrity and increased cellular stress, potentially allowing the infiltration of harmful substances, which may lead to cellular damage [25]. Additionally, increased vacuolation and changes in intracellular substances suggested increased autophagy or lysosomal activity as a cellular response to manage and restore damaged cellular components [27]. Blood vessel spasms and sequential nuclear breakdown further suggest severe cellular stress and potential disruption of blood flow and nutrient delivery, which could be the cause of the observed cellular damage. The immunofluorescence analysis revealed significant alterations in the immune cell profiles between the control and NH_3_-treated groups. The increased clustering and number of CD8^+^ cells, and to a lesser extent CD4+ cells, in the NH_3_-treated group indicate an active immune response to NH_3_ exposure. This suggests an ongoing inflammatory response, as CD8^+^ cytotoxic T cells are typically involved in responding to cellular stress and damage [28]. These findings are consistent with previous studies that have demonstrated the immunomodulatory effects of environmental toxins, including NH_3_, which can alter the distribution and function of immune cells [29]. The changes in cell density and distribution observed through DAPI staining further suggest areas of increased cellular activity, potentially due to immune cell infiltration and proliferation in response to NH_3_-induced stress.

Toll-like receptors (TLRs) are membrane-bound receptors primarily found on immunological and epithelial cells. The TLRs assist NOD-like receptors (NLRs) in activating NLRP3, leading to the production of proinflammatory cytokines. There is a known connection between diseases affecting inflammasomes and various immunological diseases, such as Muckle-Wells Syndrome, Familial Cold Autoinflammatory Syndrome, and Neonatal-Onset Multisystem Inflammatory Disease, among others [30, 31]. However, no research has been conducted to establish the mechanism of NLRP3 inflammasome activation in chicken lymphoid organs through NH_3_ exposure. The molecular analysis of mRNA and protein levels of key inflammatory genes and proteins further elucidated the mechanisms underlying the observed cellular and immunological changes. The significant upregulation of TLR-7, MYD88, NF-κB, and other related genes in the NH_3_-treated group highlights the activation of the TLR-7/MyD88/NF-κB signaling pathway in the thymus and BF of chickens. This pathway is crucial for initiating inflammatory responses and has been implicated in various forms of cellular stress and immune responses. The observed degradation of IκBα and increased levels of NF-κB (p65) and its phosphorylated form (p-p65) indicate the activation of NF-κB, a key transcription factor in the inflammatory response. This activation is consistent with the upregulation of NLRP3 inflammasome components, including NLRP3, Caspase-1, and IL-1β, further supporting the role of NH_3_ in inducing a robust inflammatory response through the activation of these pathways [37, 38]. The TLR7-MYD88-NF-κB signaling pathway is critical for activating inflammasomes in chicken BF and thymus tissues following exposure to NH_3_. The exposure to NH_3_ may also disrupt immunological homeostasis, leading to inflammasome activation via the TLR7-MYD88-NF-κB pathway in these tissues. Previous studies have reported the mechanisms by which ammonia disrupts immunological homeostasis through oxidative stress, mitochondrial dysfunction, and variation in energy metabolism and various biochemical parameters (*e.g.*, alkaline phosphatase, aspartate aminotransferase, alanine aminotransferase, and gamma-glutamyl transferase) [32-34]. Consequently, NH_3_-exposed chickens exhibited increased inflammatory responses and tissue damage. These findings indicate that NH_3_-induced activation of the TLR-7/MyD88/NF-κB pathway and NLRP3 inflammasome may result in immunological dysregulation and inflammatory diseases in chickens. Further molecular investigation is needed to elucidate the underlying mechanisms of immunological dysregulation and inflammasome activation in chicken BF and thymus tissues.

Overall, these findings suggest that NH_3_ exposure significantly impacted the cellular and immunological landscape of lymphoid organs in broilers. The activation of inflammatory pathways and the resulting cellular damage emphasize the importance of maintaining a healthy environment to mitigate the toxic effects of NH_3_ exposure. While the preliminary investigations in this study show the activation of transcription factors upon NH_3_ exposure, particularly implicating the NF-κB pathway, further studies should identify the specific transcription factors and protein targets, such as through chromatin immunoprecipitation sequencing and RNA sequencing directly activated upon NH_3_ exposure. Additionally, the proteins and biomolecules that show no changes or are downregulated upon NH_3_ exposure need to be identified, such as through proteomic and transcriptomic techniques, to explore the NH_3_-induced specific biological effects.

## Conclusion

This study exclusively demonstrated that NH_3_ exposure resulted in significant immunological damage in the lymphoid organs of broiler chickens, primarily via the activation of the TLR-7/MyD88/NF-κB signaling pathway and the NLRP3 inflammasome. The study also revealed structural impairments in the bursa of Fabricius and thymus, characterized by mitochondrial swelling, chromatin condensation, and blood vessel spasms. Immunofluorescent analysis indicated an increased immune response, evidenced by the clustering of CD4^+^ and CD8^+^ cells in the affected tissues after exposure to NH_3_. Additionally, the study observed upregulated mRNA and protein expressions of TLR-7, MYD88, and NF-κB, confirming the involvement of these molecules in the inflammatory response to ammonia exposure. The robust activation of key inflammation-related genes and proteins highlights the important role of the TLR-7/MyD88/NF-κB pathway in mediating the immune dysregulation caused by NH_3_. These findings provide a deeper understanding of the molecular mechanisms underlying NH_3_-induced immunotoxicity in chickens, offering potential avenues for developing targeted interventions to mitigate adverse effects of NH_3_ in poultry, thereby improving overall poultry health and productivity. Further studies are warranted to identify the specific transcription factors and protein targets directly activated upon NH_3_ exposure. Additionally, the proteins and biomolecules that show no changes or are downregulated upon NH_3_ exposure need to be identified to unveil the explicit biological effects induced upon exposure to NH_3_.

## Figures and Tables

**Table 1:** Primers Used in qRT-PCR.

S. no	Gene	Forward primer (5’-3’)	Reverse primer (3’-5’)
1	NF-KB	CACATGGTGGTGACCGCCAATAG	GTGCCATCGTATGTAGTGCTGTCC
2	TLR-7	GAAGTGGTATGCTCTGCCTGCTG	GTCTGCGACTCCAATCTCTGTTCC
3	TRAF6	CCTAATCGCCGTGGCCTTCTTAAC	GGAGGAGGTAGATGGTCGGATTGG
4	NLRP3	GCTCCTTGCGTGCTCTAAGACC	TTGTGCTTCCAGATGCCGTCAG
5	IL-1β	CAGCCGTCCTCCTCCGTCAC	AGCAGCCTCAGCGAAGAGACC
6	IL-18	AGATGATGAGCTGGAATGCGATGC	ATCTGGACGAACCACAAGCAACTG
7	MDY88	AAGGTGTCGGAGGATGGTGGTC	GGAATCAGCCGCTTGAGACGAG
8	β-actin	CCAGCCATGTATGTAGCCATCCAG	ACGGCCAGCCAGATCCAGAC
